# Apoptotic and anti-migratory activity of *Citrus limon* rind essential oil encapsulated in niosome against human breast cancer cells

**DOI:** 10.22038/AJP.2024.24656

**Published:** 2024

**Authors:** Mohammad Mehdi Yaghoobi, Toktam Deylami, Masoud Torkzadeh-Mahani

**Affiliations:** 1 *Department of Biotechnology, Institute of Science and High Technology and Environmental Sciences, Graduate University of Advanced Technology, Kerman, Iran*

**Keywords:** Nutrition, Anticancer plant, Limonene, Drug delivery

## Abstract

**Objective:**

This study aimed to formulate *Citrus limon* fruit rind’s essential oil (EO) and to analyze its impact on breast cancer cells.

**Materials and Methods:**

Initially, the cytotoxicity of EO (0.09-9 µg/ml) and doxorubicin (0.5-5 µM) on MCF-7, SK-BR-3, and MDA-MB-231 breast cancer cells was assessed using the MTT assay following 24-72 hr treatment. The cells were then treated with niosomes containing 4.5 µg/ml EO and 0.5 µM doxorubicin for 48 hr. Flow cytometry, migration assay, and RT-qPCR were used to study the cell behavior.

**Results:**

MTT results indicated that both EO and doxorubicin alone exhibited severe cytotoxicity (viability ≤ 30%) towards all three cells after 48 hr . When treated with encapsulated EO, the apoptotic death rate in MCF-7, MDA-MB-231, and SK-BR-3 cells was substantially diminished to 14%, 28%, and 9%, respectively. Similarly, encapsulated doxorubicin induced mild apoptotic death in these cells. Encapsulated EO and doxorubicin significantly prevented the migration of all cells. Following treatment with the encapsulated EO, a notable reduction in the expression of *VIM*, *SLUG*, *SNAIL*, and *JUN*, which are promoters of cancer cell invasion, was seen. Conversely, the expression of the FAS receptor, an active participant in the extrinsic apoptosis pathway, increased (p˂0.01).

**Conclusion:**

Lemon EO has apoptotic and anti-migratory effects on breast cancer cells, comparable to that of doxorubicin. Utilizing niosome as an efficient delivery modality effectively mitigates the adverse effects of EO and can be used for *in vivo* delivery of it to cancer cells.

## Introduction

In 2020, breast cancer was found to have the highest global prevalence (Sung et al., 2021). Breast cancer is commonly treated with chemotherapy, hormone therapy, radiation therapy, and surgery. However, for triple-negative breast cancer, chemotherapy is the only effective treatment. Multidrug resistance is a challenge in breast cancer treatment, reducing the effectiveness of drugs like doxorubicin and paclitaxel (Ji et al., 2019). Natural plant compounds have shown promise in overcoming this resistance by targeting specific genes or pathways. These compounds can also be used alongside conventional drugs to reduce their side effects. Some plant compounds even have direct anticancer properties, making them valuable in the fight against breast cancer. Secondary plant metabolites interact with transcription factors, kinases, and growth factor receptors, halting the cell cycle, triggering apoptosis, and activating tumor-suppressing genes. Importantly, these plant compounds have lower adverse effects and toxicity than synthetic pharmaceuticals (Jiang et al., 2013). Identifying such compounds in plants and detecting their target pathways in cancer cells makes it possible to optimize their use and achieve combined therapy with fewer side effects rather than relying on inefficient methods (Li et al., 2017; Mitra and Dash, 2018; Newman and Cragg, 2020).

Lemon (*Citrus limon *L.) from the Rutaceae family is among the plants studied. Polyphenol compounds are present in citrus fruits, which encompass a diverse range of bioactive compounds such as flavonoids and limonoids. Notably, the peel and fruit of lime contain substantial quantities of phenolic compounds and flavonoids. A large part of the biologically valuable compounds of lemon, including its essential oils, are in its rind, which is not edible (Mahato et al., 2018). The use of *C. limon* fruits as a remedy against many diseases is recorded in the earliest material in the Mediterranean region and the Middle East (Arias and Ramón-Laca, 2005). In Iranian traditional medicine, citrus fruit and peel have been used to treat indigestion, inflammation, and infections. Fresh lemon fruit extract with garlic (*Allium sativum* L.) is recommended in Iranian traditional medicine for treating different tumors. Also, lemon extract syrup and honey are recommended to strengthen the immune system (Arias and Ramón-Laca, 2005; Motavalizadeh Ardekani et al., 2012). Citrus essential oil, known for its antioxidant, antidiabetic, antifungal, antibacterial, and insecticidal properties, has found numerous applications in the pharmaceutical, cosmetic, agricultural, and food industries (González-Mas et al., 2019; Mahato et al., 2018). Moreover, citrus flavonoids play a crucial role in safeguarding against the harmful effects of ultraviolet rays from the sun and exhibit potent antibacterial properties (Sharma, Mahato and Lee, 2018). Lemon peel flavonoids, on the other hand, possess anti-cancer properties and the ability to impede the cell cycle, reduce inflammation, inhibit angiogenesis, hinder proliferation, suppress metastasis, and induce apoptotic cell death (M’hiri et al., 2017). Interestingly, the anticancer property of citrus rind extract is higher than pure single compounds, indicating the synergistic effect of these compounds (Koolaji et al., 2020). Besides, some essential oils synergize if combined with drugs such as doxorubicin (Zare et al., 2021). A quantitative systematic study in 2013 showed that a high intake of citrus fruits reduces the risk of breast cancer by 10% (Song and Bae, 2013). 

Because essential oils are insoluble in water, they are better delivered to cells with the help of carriers such as niosomes. Niosomes prevent the sudden release of compounds and gradually deliver them to target cell. Utilizing niosomes has the advantage of increasing agent's solubility, availability, and stability (Asbahani et al., 2015). Using drug delivery methods like niosomes to administer plant compounds yields better results than releasing them suddenly. In contrast, delivering compounds without a carrier can cause severe cytotoxic effects on cells *in vitro*, which is scientifically unacceptable and not applicable in living organism models. Besides enabling the controlled release of medicinal and essential oil constituents to the cell, niosomes can also aid in the delivery of certain compounds that are poorly soluble in water into the cell (Yeo et al., 2018). 

Plants' medicinal properties and secondary metabolites vary based on species, location, and climate. Therefore, their effects on cancer cells may also differ, requiring the study of plant compounds in each region. The impact of essential oil derived from native Iranian lemon on breast cancer cells using niosomes nanocarrier remains unexplored. Therefore, this study aims to investigate the effects of citrus rind essential oil in the niosome against three breast cancer cell lines, namely MCF-7, MDA-MB-231, and SK-BR-3.

## Materials and Methods

### Essential oil extraction and analysis

**Figure 1:**
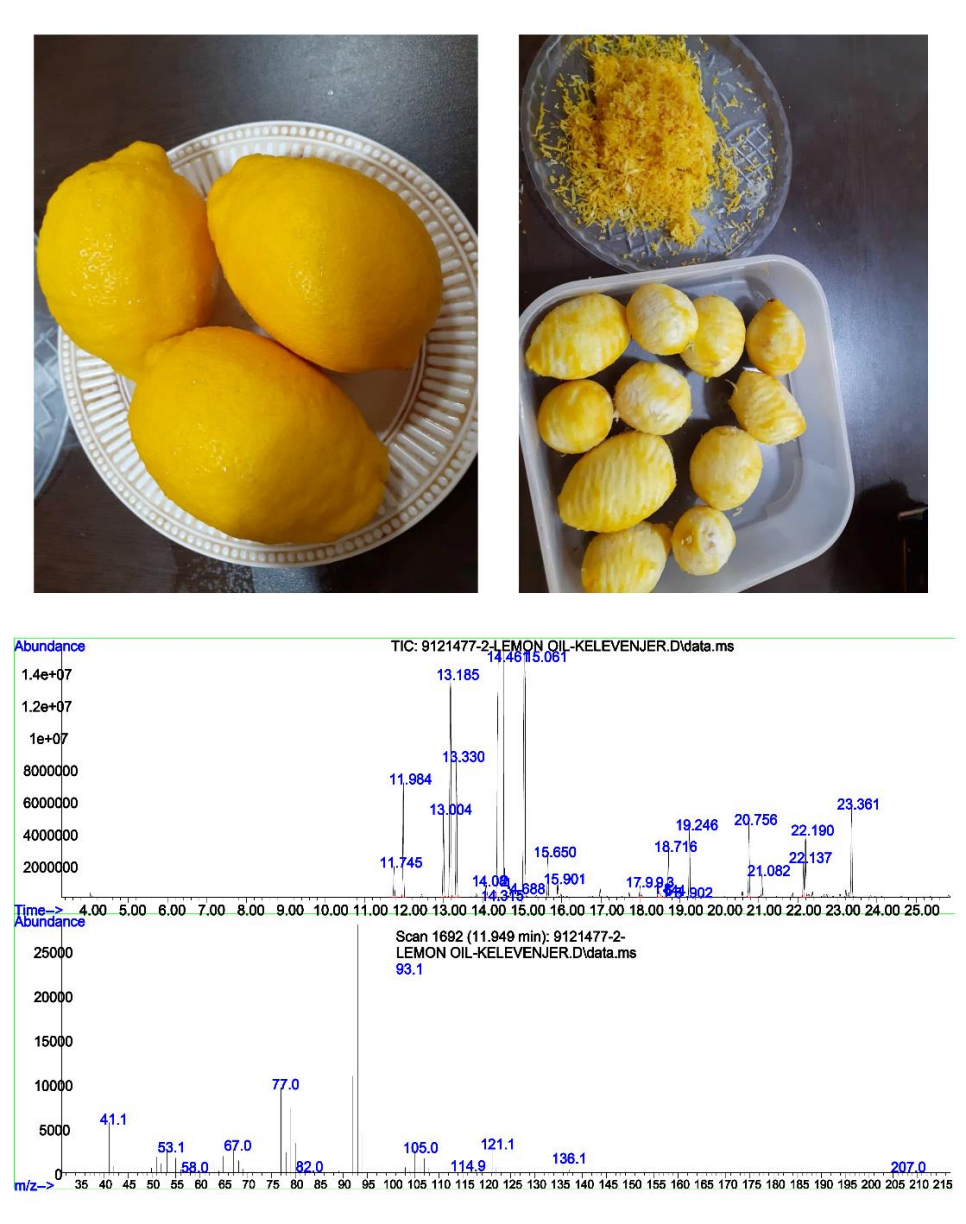
Images of lemon fruits before and after shredding the epicarp, along with the GC-MS results of the EO.

The Eureka variety of *Citrus limon* (L.) Osbeck, cultivated in a large area in Jahrom city, Iran, was prepared in February 2021. The fruit of this variety has a thick yellow skin and seeds and it possesses an elongated shape with an oval longitudinal section. Only intact fruits that lacked any spots indicative of disease were selected and washed with water. Then, the epicarp (flavedo) of the rind was removed using a shredder while ensuring that the mesocarp (albedo) was not affected (Figure 1). The grated sample was poured into a container containing distilled water to prevent the evaporation of the volatile oils. Subsequently, essential oil (EO) was extracted from 100 g of grated skin with European Pharmacopoeia Clevenger apparatus and dehydrated using sodium sulfate powder. The EO was stored in a sterile screw-capped tube with a dark coating and refrigerated (ISO, 1996). A sample of the EO was dispatched to Raymon Dana Dayan's laboratory in Tehran for compound identification using the Agilent 5975C GC-MSD apparatus. Essential oil compounds were identified based on their retention time and exit from the device. In accordance with established standards, their percentages in the total essential oil were documented.

A sample of dried lemon rind was sent to Marjaan Khatam Company in Tehran for pesticide residue analysis by GC-MS and LC-MS-MS methods. Pesticide residues were measured by quantifying 78 different types of common pesticides as mg/kg. The results reported as permissible limit or exceeding it, as per international standards. The interaction between EO compounds and breast cancer cell proteins was investigated through the Stitch website (http://stitch.embl.de/).

### Culture of breast cancer cells

MCF-7, MDA-MB-231, and SK-BR-3 cells were provided from the National Cell Bank of the Pasteur Institute of Iran (Tehran, Iran) and cultured in Dulbecco's Modified Eagle Medium (DMEM) with 8% fetal bovine serum (FBS) (both from Thermo Fisher Scientific, USA) within a cell culture incubator at 37°C with 5% CO_2_. 

### Measuring the effect of EO on cells by MTT assay

In each well of a flat-bottomed 96 microplate, a total of 10,000 SK-BR-3 cells and 15,000 MCF-7 and MDA-MB-231 cells were cultured using 100 µl of complete culture medium. Upon reaching 60-70% confluency, fresh medium containing 0.09-9 µg/ml of EO was added to wells of each treatment group. A positive control was included using 0.5-5 µM doxorubicin (EBEWE Pharma, Austria). Four replicates were performed for each treatment in a column, while cells in another column were cultured in the standard medium as a control group. After 24, 48, and 72 hr of treatment, MTT solution (5 mg/ml in PBS) was added to each well, followed by Dimethylsulfoxide solvent. Absorbance was measured at 570 nm using an ELISA Reader (USA, Biotek). IC50 was calculated in Excel software.

### Niosome synthesis and loading

As described previously, a thin film hydration technique was utilized for niosome synthesis using Span 60, cholesterol, and Tween (Barani et al., 2019).

The entrapment yield of niosome (EY) was determined by measuring the absorbance of the desired agent in 200-750 nm with a UV-Vis spectrophotometer. Doxorubicin and EO showed a typical peak at 500 and 225 nm wavelengths, respectively. The calibration curve was recorded at these wavelengths. The formulations were centrifuged, and the absorbance of the supernatant was read at the above wavelengths. The EY of EO and doxorubicin was calculated based on the following formula: 

EY%= (Total amount of all-all in the supernatant)/ Total amount of all ×100

### Measurement of cell death by Annexin V-PI staining

The cells were cultured in 60 mm culture dishes and, following reaching 60-70% confluency, were exposed to EO (4.5 µg/ml) and doxorubicin (0.5 µM) loaded in niosomes. The cells of the control group were cultured in a standard culture medium. After 48 hr of treatment, the cells were washed with the binding buffer (Annexin V apoptosis detection kit, eBioscience^TM^, Thermo Fisher Scientific, USA) and suspended with a concentration of 10^6^/ml in the binding buffer. Afterward, 5% v/v of Annexin V-FITC solution was added to the cell suspension and incubated at room temperature for 10-15 min. The cells were then washed and stained with 5 µl of PI. The cells were kept on ice and after about 30 min, were analyzed with Cyflow space (Partec, Germany).

The FL1 and FL2 histograms were used to measure FITC and PI dye emissions, respectively. Initially, unstained live cells were used to determine optimal settings. Cells were then stained with either PI or FITC alone. Once optimal settings for the FSC, SSC, FL1, and FL2 channels were determined, the cells were stained with both dyes. The resultant data were then analyzed in the Q1, Q2, Q3, and Q4 quadrants on the FL1/FL2 dot blot using the FlowJo software (FlowJo 10.5.3, 2018).

### Staining cells with eosin and methylene blue

The cells were cultured on a coverslip in a 24-well plate. The experimental groups were the same as before. After 48 hr, the cells on the coverslip were fixed in methanol. The slides were stained with eosin and methylene blue 1% (w/v) (Merck Company) for 30 sec. The coverslips were dried and mounted on a slide. The slides were photographed with a camera on a light microscope.

### Measuring the rate of cell migration by scratch test

Around 10^6^ SKBR-3, MCF-7, and MDA-MB-231 cells were placed in a 24-well plate. Once the cells were about 60-70% confluent, a scratch was made from north to south using a yellow tip to remove the cells in that area. The wells were then photographed. The same experimental groups were used as in the previous stage. After 48 hr, the cells were examined under a microscope and photographed again. The extent of the scratched surface was measured using the ImageJ program with the Wound_Healing_Size_Tool plugin (Suarez-Arnedo et al., 2020). The mean rate at which the scratched area was replenished in the groups treated with EO or doxorubicin, was compared with the control group and analyzed using GraphPad Prism.

### Evaluation of gene expression by RT-qPCR

The three cells were treated with 4.5 µg/ml EO loaded in niosomes for 48 hr. Then, total RNA was extracted using the Total RNA Extraction Kit (Jena Bioscience, Germany) according to the manufacturer's instructions. RNA concentration was measured using a spectrophotometer, and its quality was checked by agarose gel electrophoresis. cDNA was synthesized using EasyTM cDNA Synthesis Kit (Parstous, Iran) on 1 μg of total RNA digested with DNase I (Thermo Scientific, USA). Two negative controls without enzyme and template were also used in each run.

The RT-qPCR volume included 2X RealQ Plus Master Mix Green solution (Amplicon, Denmark), one μl of cDNA diluted at 1:10, and 150 nmol of each primer (Table 1) in a final volume of 20 μl. The temperature program consisted of 95°C for 15 min, 40 cycles of 95°C for 30 sec, 59°C for 30 sec, 72°C for 30 sec, and 80°C for 5 sec in Rotor-Gene 3000 machine (Corbett Research, Australia). Raw fluorescence data were collected and exported to LinRegPCR software (v 2021) (Ruijter et al., 2009). The initial concentration was normalized against the reference gene (*GAPDH*). The cDNA of the untreated cell was selected as a calibrator. Primers were designed using the Primer-BLAST database and were ordered from SinaClon Company (Tehran, Iran).

The relationship between gene expression and survival of breast cancer patients was investigated in the Kaplan-Meier plotter database: https://kmplot.com/analysis/index.php?p=background

### Statistical analysis

The quantitative variables of cell viability, scratched area, gene expression, and cell death rates between the control and treatment groups exposed to either EO or doxorubicin, were compared by parametric tests. This analysis was conducted using the Paired Sample T-test or Two-Way ANOVA statistical methods, utilizing either Microsoft Excel 2019 or GraphPad Prism 9 software. Three replications were considered for each treatment or control group. Mean, SD, variance, and normal distribution of data were calculated in Excel programs. Data are presented as Mean±SD, and a significance level of <0.05 was considered.

## Results

### Analysis of EO from lemon rind

The results from the analysis of pesticide residues indicated that among the 78 pesticides tested, only a minute amount of 0.086 mg/kg of propiconazole was identified in the EO. No other agricultural pesticides were found in the sample.. Approximately 0.05 ml of essential oil was obtained per gram of grated epicarp, with a density of 0.895 g/cm^3^. The GC-MS results indicated that limonene (47.88%), -Terpinene (12.76%), β-Pinene (8.97%), and α-Pinene (3.44%) were the predominant compounds in the EO (Figure 1 and Table 2). 

### Measurement of EO and doxorubicin effect on the viability of breast cancer cells by MTT assay

**Figure 2:**
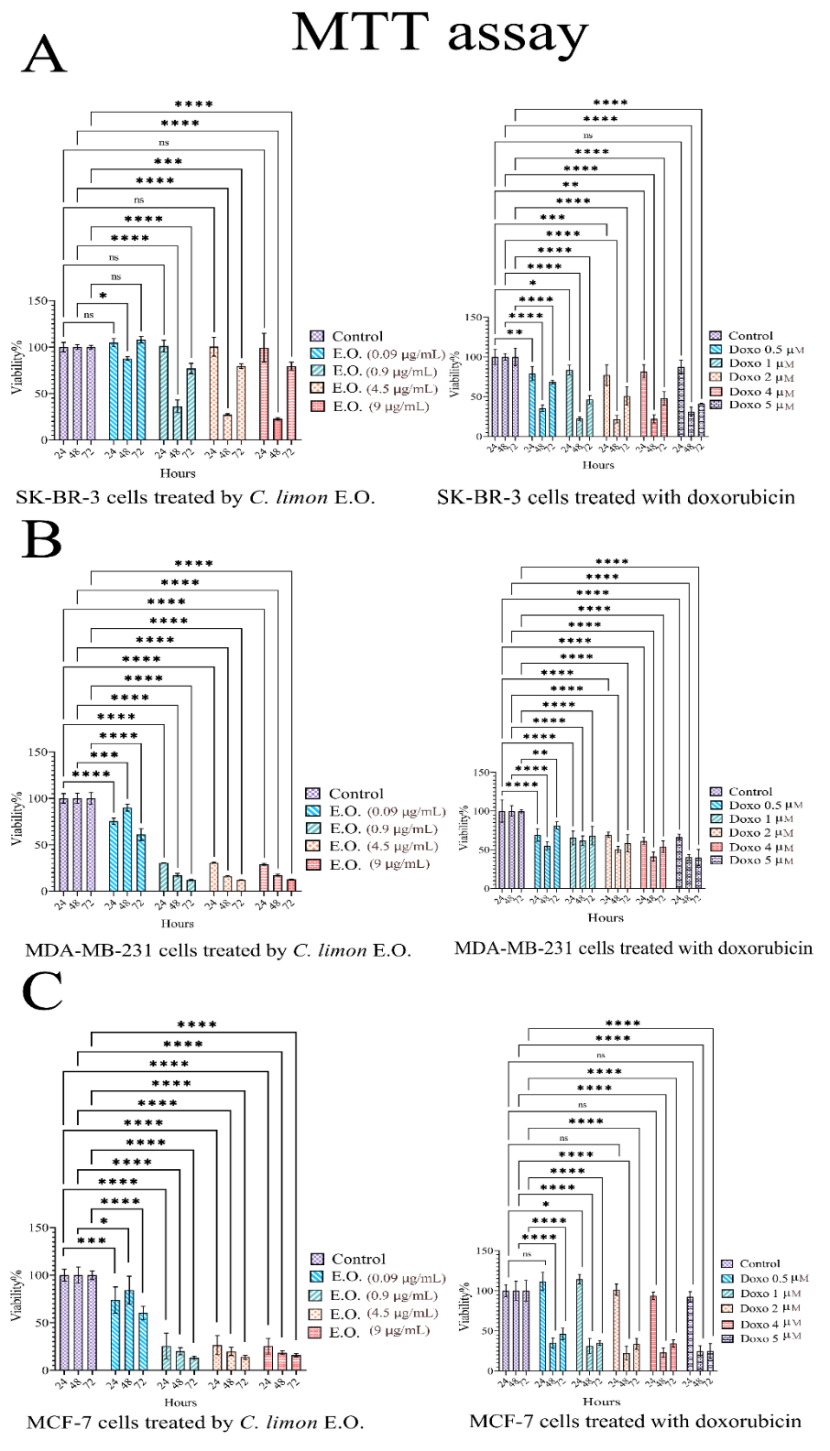
The viability of SK-BR-3, MDA-MB-231, and MCF-7 cells following treatment with EO and doxorubicin at 24, 48 and 72 hr determined by MTT assay. Data are presented as Mean±SD. (ns=non-significant, *p<0.05, **p<0.01, ***p< 0.001, and ****p<0.0001).

The viability of SK-BR-3 cells was significantly reduced to 20-80% after exposure to ≥0.5 µM doxorubicin for 24-48 hr. Additionally, treatment with 0.9 µg/ml of EO for 48 hr significantly decreased cell viability to 22-36% in comparison to the control group (p˂0.0001) (Figure 2A). The survival of MDA-MB-231 cells decreased to 40-60% following the addition of doxorubicin. Higher concentrations of EO caused a sharp decline in cell viability to <30% after 24 hr and to 13% after 72 hr in comparison to the control group (p<0.0001) (Figure 2B). Similarly, the viability of MCF-7 cells decreased significantly to <50% after 48 hr of doxorubicin exposure in comparison to the control group (p˂0.0001). Higher concentrations of EO led to a sharp decrease in MCF-7 viability to 26% after 24 hr and 13% after 72 hr in comparison to the control group (p<0.0001) (Figure 2C). The results indicate that the EO exhibited a more potent cytotoxic effect than the drug. Based on the MTT assay data, subsequent experiments utilized 0.5 µM of doxorubicin and 4.5 µg/mL of EO loaded in niosomes. The entrapment yield (EY%) of EO and doxorubicin in the niosomal formulation was 99.5% and 97.41%, respectively. IC50 values for all three cells at 24, 48, and 72 hr are presented in Table 3.

### Determining the type and rate of cell death by Annexin V/PI staining

**Figure 3:**
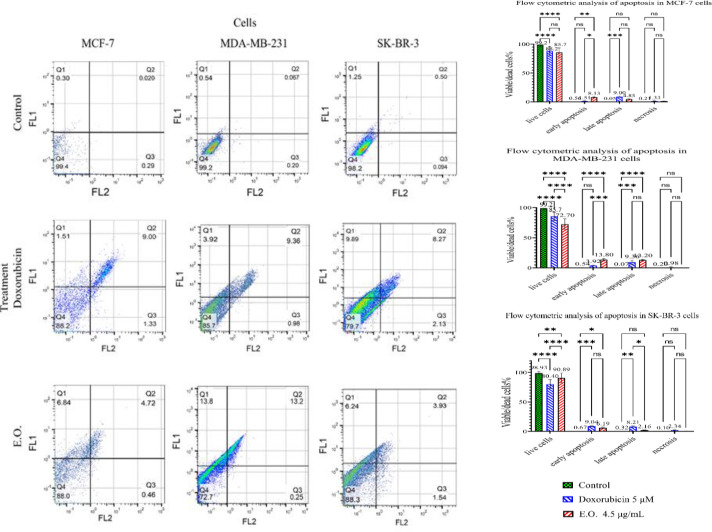
Apoptotic effects of 4.5 µg/ml EO and 0.5 µM doxorubicin loaded in niosome on MCF-7, MDA-MB-231, and SK-BR-3 cells, determined by flow cytometry. Data are presented as Mean±SD. (ns=non-significant, *p<0.05, **p<0.01, ***p<0.001, and ****p<0.0001)

The flow cytometry data revealed a significant decrease in viable MCF-7 cells from 99.20% in the control group to 88.2% and 85.7% after exposure to doxorubicin or EO, respectively (p˂0.0001 for both treatments). There was no difference in viable cell percentage between the two treatments. EO treatment resulted in 8.13% early apoptosis and 4.85% late apoptosis, while doxorubicin caused 1.51% early apoptotic death and 9% late apoptotic death (Figure 3).

Similarly, viable MDA-MB-231 cells decreased significantly from 99.20% to 85.7% and 72.7% following exposure to doxorubicin and EO, respectively. The cytotoxicity of EO was significantly higher than that of doxorubicin compared to the control (p˂0.0001). EO treatment caused 13.80% early apoptosis and 13.20% late apoptosis in MDA-MB-231 cells, while doxorubicin caused 3.92% early apoptotic death and 9.36% late apoptotic death (Figure 3). 

A significant decrease in viable SK-BR-3 cells was recorded from 98.93% to 80.40% following doxorubicin and 90.89% following EO treatment. The lethality of doxorubicin was significantly higher than EO on SKBR-3 cells (p˂0.0001). Following treatment with EO, SK-BR-3 cells underwent 6.19% early apoptosis and 2.16% late apoptosis. However, doxorubicin led to 9.04% early apoptotic death and 8.21% late apoptotic death (Figure 3). 

Treatment with 4.5 µg/ml EO for 48 hr left 16%, 20%, and 27% of MDA-MB-231, MCF-7, and SKBR-3 cells alive, respectively. However, treatment with the same amount of EO in the niosome resulted in higher cell survival rates (>72%). These data indicate that the cytotoxicity of the niosome-incorporated essential oil was comparatively milder than the cytotoxicity demonstrated by the standalone EO.

**Figure 4:**
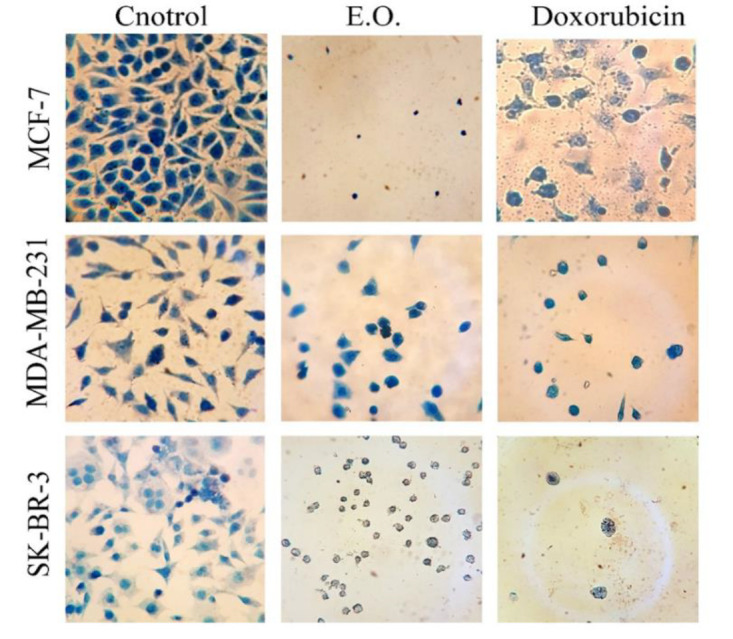
Eosin/methylene blue staining of MCF-7, MDA-MB-231, and SK-BR-3 cells before and after treatment with 4.5 µg/ml EO and 0.5 µM doxorubicin loaded in niosome (magnificationX320).

Eosin/methylene blue staining revealed that the control group cells had healthy and normal morphology with multiple nuclei. However, treatment with doxorubicin or EO caused the appearance of small, irregularly shaped, and wrinkled cells, along with debris, indicating cell death (Figure 4). 

### The rate of cell migration after treatment with EO and doxorubicin

**Figure 5:**
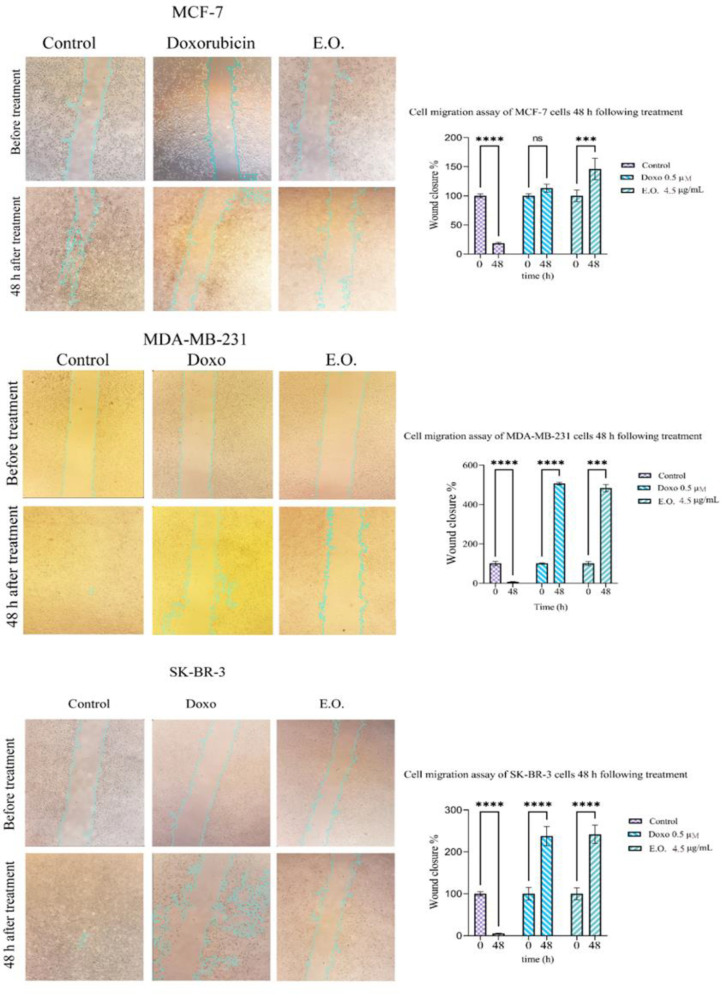
Migration assay of MCF-7, MDA-MB-231, and SK-BR-3 cells before and after treatment with 4.5 µg/mL essential oil (EO) and 0.5 µM doxorubicin (DOXO) loaded in niosome. Data are presented as Mean±SD. (ns=non-significant, ***p<0.001, and ****p<0.0001). Magnification ×100.

Figure 5 illustrates the filling rate of the scratched gap in the control and treated groups. The control group showed a significant reduction in the scratched surface after 48 hr for all three cell types, indicating cell migration from the surrounding area. Notably, MCF-7 cells exhibited less migratory and invasive behavior than the other two cell types. Specifically, MCF-7 cells had a filling rate of approximately 82%, while SK-BR-3 and MDA-MB-231 cells had 95% and >98%, respectively (p˂0.0001 for all three cell types). EO treatment significantly increased the scratched surface for all three cell types due to massive cell death compared to the control (p<0.001). Similarly, doxorubicin treatment increased the scratched surface for MDA-MB-231 and SK-BR-3 cells but not for MCF-7 cells (Figure 5).

### Evaluation of gene expression changes after treatment with EO by RT-qPCR

**Figure 6:**
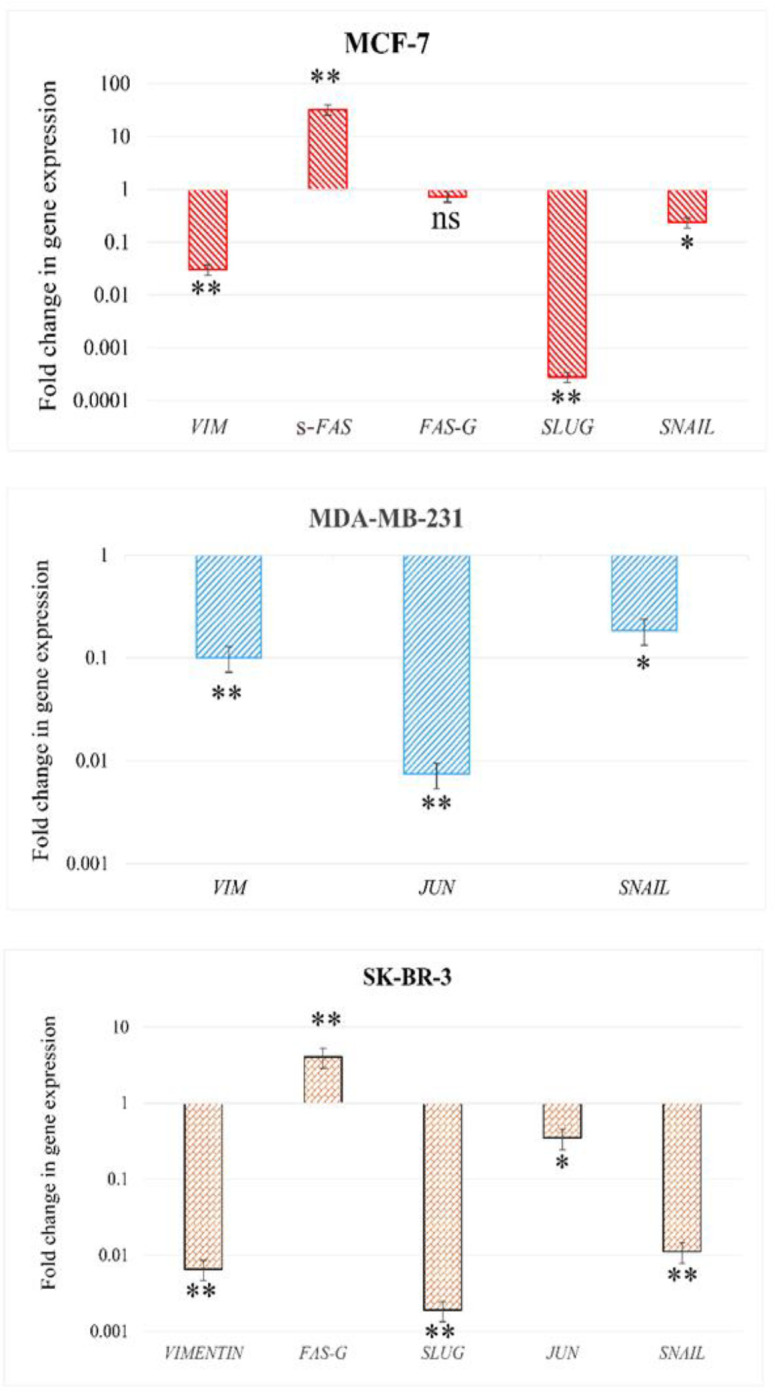
qRT-PCR data showing changes in expression of *FAS, VIM, SLUG, SNAIL*, and *JUN* 48 hr following treatment of MCF-7, MDA-MB-231, and SK-BR-3 cells with 4.5 µg/ml EO. Data are presented as Mean±SD. (ns=non-significant, *p<0.05 and **p<0.01).

In the following section of this investigation, the quantification of specific genes associated with apoptotic cell death or pathways linked to aggressiveness, was carried out after the administration of EO. The results indicated a substantial reduction in *VIM, SLUG, JUN*, and *SNAIL* gene expression in all or some of the three cell types. Conversely, there was a significant elevation in the expression of the *FAS* receptor (Figure 6).

**Figure 7:**
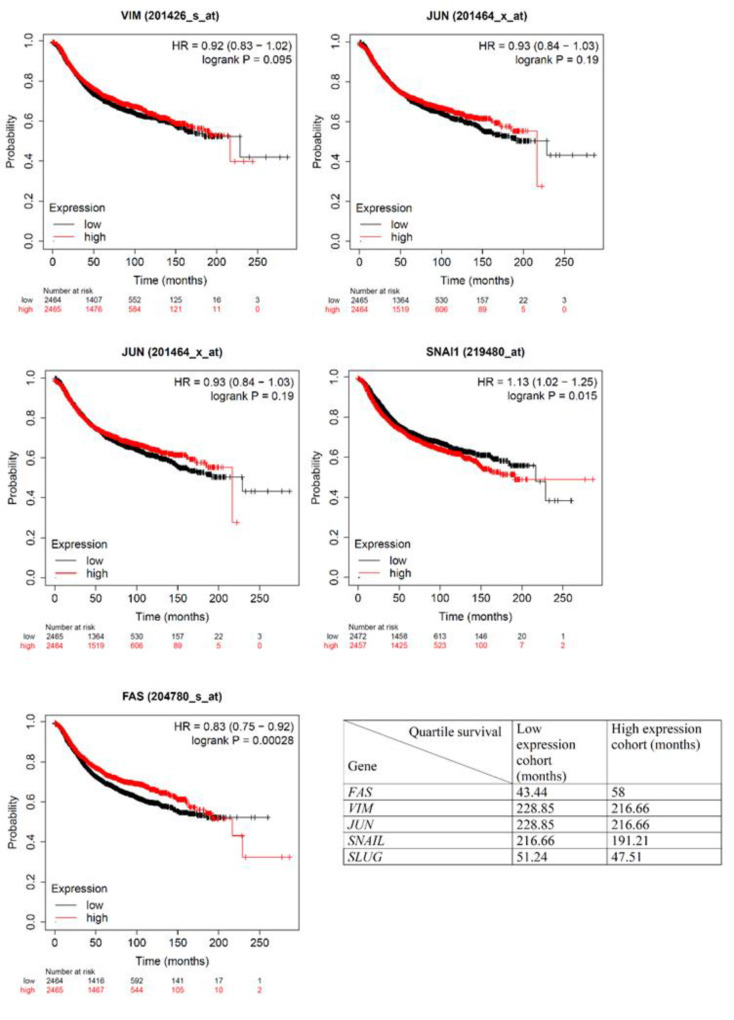
Kaplan-Meier plots showing the effect of expression of *FAS, VIM, SLUG, SNAIL*, and *JUN* genes on the survival of breast cancer patients. Data were retrieved from https://kmplot.com/analysis/index.php?p=background

**Figure 8:**
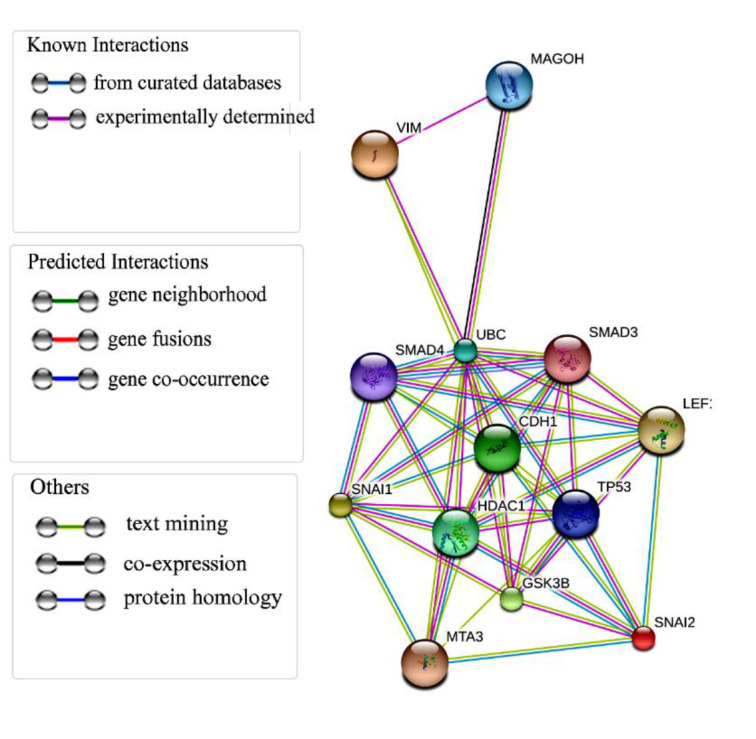
The interaction of VIM, SLUG, and SNAIL proteins with other molecules as retrieved from the Stitch website (http://stitch.embl.de/).

The Kaplan-Meier plots demonstrate that the augmentation in *FAS* expression is accompanied by an elevation in the average survival duration of patients, progressing from 43.44 to 58 months. Nevertheless, the elevated expression of the four genes, namely *VIM, JUN, SNAIL*, and *SLUG*, was correlated with a decline in the average survival duration of patients (Figure 7). Figure 8 shows the interaction of VIM, SNAI1, and SNAI2 through mediators GSK3B and ubiquitin C and their interaction with other proteins.

## Discussion

This study investigated the effects of the essential oil (EO) extracted from *C. limon* rind on three human breast cancer cells. The absence of agricultural pesticides in the lemon rind can be attributed to the non-utilization of pesticides in gardens or the prolonged interval between pesticide application and subsequent measurement of pesticide residues (Fantke et al., 2014). Therefore, any potential lethal or inhibitory attributes within the EO sample are solely because of its natural compounds, not pesticides. 

The MCF-7 cell line expresses both estrogen and progesterone receptors while lacking the HER2 receptor. This particular cell line is commonly employed in the study of anti-cancer drugs and the exploration of drug resistance mechanisms. Conversely, SK-BR-3 cells do not express estrogen and progesterone receptors but do exhibit the HER2 receptor. SK-BR-3 cells are primarily utilized in research on drugs that specifically target the HER2 receptor. On the other hand, MDA-MB-231 cells are known as triple negative and do not possess any of the aforementioned receptors, and are characterized by their high invasiveness. These cells are extensively employed in studies focusing on metastasis. The selection of these three cell lines was made to investigate the impact of essential oils on three distinct types of breast cancer (Dai et al., 2017).

Limonene (C_10_H_16_) was the most abundant compound in the lemon EO. A study was conducted on breast cancer patients who received a daily dose of two grams of limonene for six weeks before surgery. Limonene levels were measured in the blood and breast tissue of patients, revealing a significant accumulation in the breast tissue. This accumulation led to a decrease in cyclin D1 expression in tumor tissue. Furthermore, limonene can reduce tumor size, number, and weight or induce apoptosis *in vivo* (Miller et al., 2013; Zhou et al., 2021). The second most-abundant compound in the EO sample was γ-Terpinene, which has been reported to possess antimicrobial, antioxidant, anti-inflammatory, and antiproliferative properties (Klimek-Szczykutowicz et al., 2020). However, a lack of research on its effects on cancer cells necessitates further investigation. The observed effects of citrus EO cannot be attributed to a single compound, and there is a synergistic effect (Koolaji et al., 2020). Combination therapy represents a sophisticated strategy in the treatment of cancer. Essential oils are more potent and have more durable effects on cancer cells than their purified single compounds (Blowman and Magalhães, 2018; Koolaji et al., 2020). 

Flow cytometry revealed that the EO was more effective than doxorubicin against MDA-MB-231 cells. Conversely, doxorubicin was more potent and caused higher rates of apoptosis in SK-BR-3 cells than the EO. On the other hand, the EOhad a slower mode of action and caused early apoptotic death in MCF-7 cells, while doxorubicin caused late apoptotic death. The cytotoxicity of the EO was reduced when encapsulated, possibly due to the niosome controlling its release. It indicates the efficient function of niosome formulation for controlled release and preventing the sudden release of EO. The MTT assay data indicated that direct administration of the EO caused significant cytotoxicity. The gradual release of the oil by the niosome increased its bioavailability, which is beneficial for *in vivo* studies. The observed variance in the impact of doxorubicin and EO on the cells under investigation may also be attributed to inherent differences in the cells themselves. The MCF-7 cell belongs to the luminal A subset, expresses estrogen and progesterone receptors (PR+, ER+), and exhibits lower invasiveness. Conversely, the MDA-MB-231 cell is highly aggressive and does not express ER, PR, or HER2. The SK-BR-3 cell is a HER2+ cell and serves as an intermediary between the two cell lines regarding behavior (Dai et al., 2017). Interestingly, we observed more potent effects of EO on the highly aggressive MDA-MB-231 cells.

The study's findings indicate that both EO and doxorubicin effectively impede the migration of MDA-MB-231 and SK-BR-3 cells, surpassing the inhibition observed in MCF-7 cells (Figure 5). The gene expression analysis corroborates the migration-inhibiting effects of EO. The less aggressive nature of MCF-7 cells resulted in comparatively lower inhibition of their migration and invasion. Elevated *VIM* expression indicates aggressiveness and poor prognosis in breast cancer patients (Wang et al., 2020). Suppression of *VIM* expression hinders carcinoma cells' attachment and migration. *SLUG* and *SNAIL* increase *VIM* and drive breast cancer cells toward metastasis (Grzegrzolka et al., 2015; Zhang et al., 2022). They also possess antiapoptotic properties. Reducing their expression or activation inhibits invasion ability and promotes apoptosis. We found that EO compounds effectively decrease the expression/activation of *VIM*, *SLUG*, and *SNAIL*, inhibiting migration and invasion of breast cancer cells. 

The extrinsic apoptosis pathway is activated by the binding of FAS ligand to FAS receptor. FAS receptor has five isoforms, including a soluble isoform (sFAS) lacking the transmembrane domain. Reports suggest that cancer cells may express sFAS to evade apoptosis (Cheng et al., 1994). Treatment of SK-BR-3 cells with EO increased expression of general FAS isoform, while MCF-7 cells showed increased sFAS. Our findings indicate that overexpression of sFAS results in higher rates of apoptosis rather than its suppression in MCF-7 cells. Similar to our results, previous studies show that some plant compounds increase proapoptotic gene expression, such as FAS and BAX, and decrease antiapoptotic gene expression, such as BCL2. The apoptotic effect of lemon extract on MCF-7 cancer cells has been reported (Alshatwi et al., 2011; Gan et al., 2020).

The hyperactivation of the JUN oncogene has made it a critical therapeutic target in breast cancer. JUN promotes the expression of cyclin D1, facilitating cancer cell proliferation (Brennan et al., 2020; Jiao et al., 2010; Luo et al., 2020). We found a significant decrease or cessation of *JUN* expression in all three cell types treated with EO. This finding is consistent with previous reports that limonene reduces cyclin D1 expression (Miller et al., 2013).

Interestingly, the Kaplan-Meier curve supports our gene expression findings. Consumption of lemon compounds, particularly its EO, may have comparable effects on gene expression in patients, potentially increasing their survival. It must be acknowledged that this study has certain limitations, including the inability to purify EO compounds and investigate their biological effects on cells, as well as the inability to conduct animal model studies. 

The present study has demonstrated the efficient encapsulation and delivery of EO of lemon rind to three breast cancer cells using niosomes. These compounds have exhibited significant cytotoxic effects on breast cancer cells and have been found to effectively prevent their migration and invasion, with potency comparable to that of doxorubicin. These findings underscore the potential anticancer properties of lemon rind, which is typically discarded and not consumed. Due to the modulation of the lethal effects of the EO by niosome, it is plausible to propose niosome as a efficient approach and a promising strategy for delivering anticancer agents to breast cancer tissues.

**Table 1:** The primer sequences of the studied genes and the amplicon’s length

Name	F primer	R primer	Length
*GAPDH*	GAGTCAACGGATTTGGTCG	GAATTTGCCATGGGTGGA	149
general *FAS*	TCAAGGAATGCACACTCACC	CCTTTCTGTGCTTTCTGCATG	153
soluble *FAS*	GGGTGGCTTTGTCTTCTTC	GGAGATTCATGAGAACCTTGG	116
*SLUG*	AGAAGCATTTCAACGCCTCC	TCTGGTTGTGGTATGACAGG	115
*JUN*	GCCAACTCATGCTAACGCAG	CTCTCCGTCGCAACTTGTC	126
*SNAIL*	CGAGTGGTTCTTCTGCGCTA	GGGCTGCTGGAAGGTAAAC	160
*VIM*	GGACCAGCTAACCAACGACA	AAGGTCAAGACGTGCCAGAG	178

**Table 2:** The composition of essential oil of Lemon rind determined by GC-MS analysis. The compounds are sorted according to their abundance.

**Compound**	**R.T.**	**Total %**
Limonene	14.461	47.88%
γ-Terpinene	15.061	12.76%
β-Pinene	13.185	8.97%
β-Pinene	13.33	4.37%
α-Pinene	11.98	3.44%
Sabinen	13	2.70%
β-Bisabolene	23.361	2.70%
Nerol acetate	20.756	2.04%
α-Citral	19.246	1.98%
α-Bergamotene	22.19	1.77%
β-Citral	18.716	1.34%
Terpinolene	15.65	1.15%
Caryophyllene	22.137	1.03%
cis-Geraniol	18.454	1.02%
Geranyl acetate	21.082	0.66%
Terpinolene	14.08	0.54%
β-Phellandrene	14.548	0.45%
o-Cymene	14.339	0.42%
Linalool	15.901	0.42%
β-Ocimene	14.688	0.36%
alpha-Terpineol	17.993	0.35%
cis-α-Bisabolene	23.216	0.30%
Citronellal	16.979	0.24%
α-Bisabolol	26.101	0.19%
Citronellol acetate	20.587	0.18%
cis-β-Farnesene	22.365	0.17%
Valencene	23.291	0.16%
(-)-4-Terpineol	17.713	0.15%
α-Phellandrene	13.832	0.14%
trans-α-Bergamotene	21.863	0.14%
n-Nonanal	15.982	0.11%
E)-β-Famesene	23.064	0.11%
Humulene	22.732	0.10%
(-)-Perillaldehyde	19.561	0.09%
Camphene	12.45	0.08%
β-Santalene	22.656	0.08%
cis-β-Terpineol	15.359	0.07%
α-Longipinene	22.895	0.06%
Limonene oxide	16.781	0.05%
Decanal	18.04	0.05%
cis-Verbenol	17.527	0.04%
cis-sesquisabinene hydrate	23.95	0.04%
Nerolidol	24.119	0.04%
Methyl geranate	20.144	0.03%
Tetradecane	21.45	0.03%
Geranyl n-propionate	22.586	0.03%
δ-Cadinene	23.6	0.03%
trans-3-Tetradecene	21.316	0.02%
α-Santalene	21.992	0.02%
Aromandendrene	23.758	0.02%
Total		100%

**Table 3:** The IC_50_ value on three cancer cell lines after 24, 48, and 72 hr of treatment with 4.5 µg/ml of EO and 0.5 µM doxorubicin

**MDA-MB-231**		**SK-BR-3**		**MCF-7**		**Cell** **Time**
**Doxorubicin IC**_50_	**EO IC**_50_	**Doxorubicin IC**_50_	**EO IC**_50_	**Doxorubicin IC**_50_	**EO IC**_50_	
7.421	0.383	40.389	156.59	12.695	2.9177	24 hr
3.053	0.90	0.652	3.5442	0.914	2.67605	48 hr
3.845	0.47	3.258	18.4549	1.622	1.35145	72 hr
